# Supplementary material for: A Novel Thiophene-Based Fluorescent Chemosensor for the Detection of Zn2+ and CN−: Imaging Applications in Live Cells and Zebrafish
Source: Sensors (Basel). 2019 Dec 11;19(24):5458. doi: 10.3390/s19245458 (PMC6961029; doi:10.3390/s19245458)
Supplement: Supplementary file 1 [file sensors-19-05458-s001.pdf]

Supporting Information

A Novel Thiophene-Based Fluorescent Chemosensor for the Detection of Zn^{2+} and CN^{-}: Imaging Applications in Live Cells and Zebrafish

Min Seon Kim [1], Dongju Yun [1], Ju Byeong Chae [1], Haeri So [1], Hyojin Lee [2], Ki-Tae Kim [2,*], Mingeun Kim [3], Mi Hee Lim [3] and Cheal Kim [1,*]

[1] Department of Fine Chemistry, Seoul National University of Science and Technology, Seoul 01187, Korea; dltmf2303@naver.com (M.S.K.); juju9593@hanmail.net (D.Y.); ch920812@naver.com (J.B.C.); gofl0988@naver.com (H.S.)

[2] Department of Environmental Engineering, Seoul National University of Science and Technology, Seoul 01187, Korea.; hyojin_lee@seoultech.ac.kr

[3] Department of Chemistry, Korea Advanced Institute of Science and Technology, Daejeon 34140, Korea; mingeun@unist.ac.kr (M.K.); miheelim@kaist.ac.kr (M.H.L.)

* Correspondence: ktkim@seoultech.ac.kr (K.-T.K.); Tel: +82-2-970-6642; chealkim@snut.ac.kr (C.K.); Tel: +82-2-971-6680; Fax: +82-2-971-9147

Table S1. Examples of chemosensors for detecting both Zn^{2+} and CN^{-}.

No.	Sensor	Detection Limit for Zn^{2+} (μM)	Detection Limit for CN^{-} (μM)	Imaging	Ref.
1	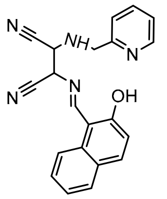	0.11 μM	No	None	[33]
2	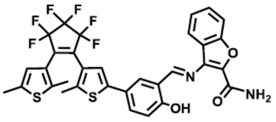	0.032 μM	0.013 μM	None	[34]
3	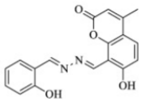	0.061 μM	0.169 μM	None	[35]
4	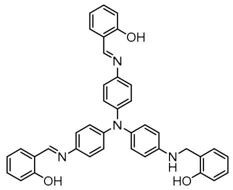	0.014 μM	5 μM	Living cells	[36]

5	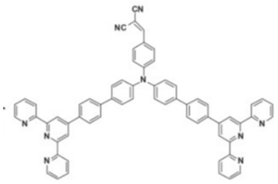	0.37 μM	3.87 μM	Living cells	[37]
6	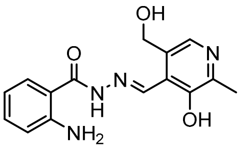	0.320 μM	0.153 μM	Living cells	[38]
7	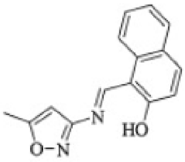	1.29 μM	12.3 μM	Living zebrafish	[39]
8	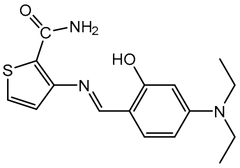	2.55 (±0.05) μM	44.6 (±1.5) μM	Living cells and zebrafish	This work

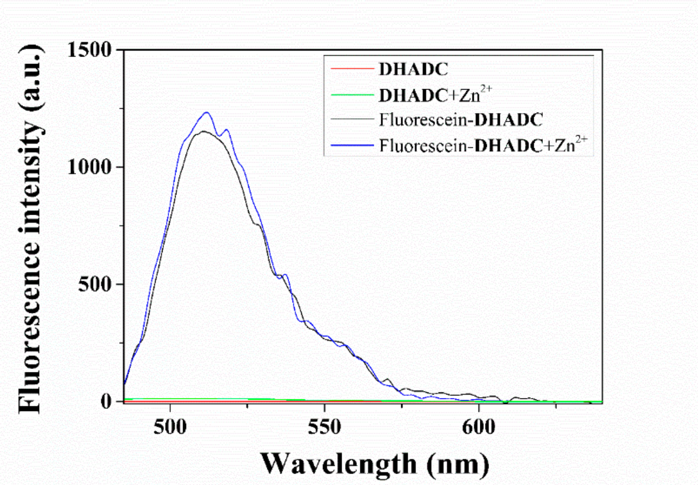

(a)

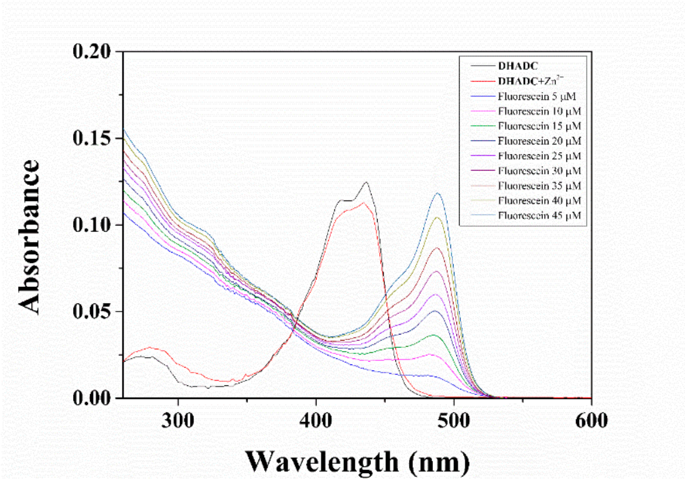

(b)

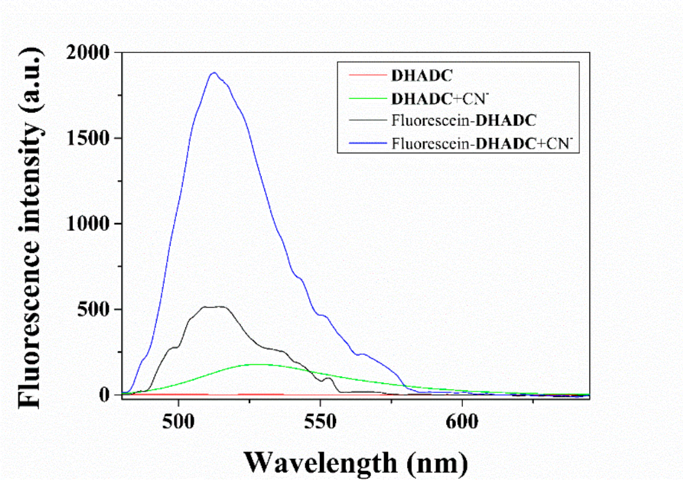

(c)

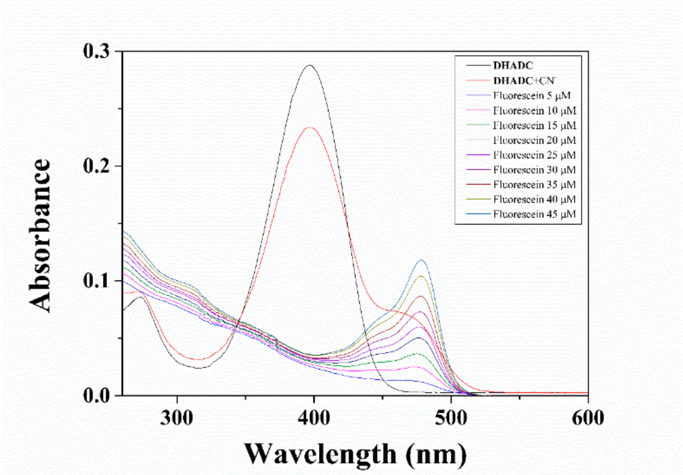

(d)

Figure S1. (**a**) Fluorescent (λ_{ex} = 464 nm) and (**b**) absorption spectra of **DHADC** (3 μM), **DHADC**+Zn^{2+} (140 equiv.) and fluorescein. (**c**) Fluorescent (λ_{ex} = 459 nm) and (**d**) absorption spectra of **DHADC** (10 μM), **DHADC**+CN^- (100 equiv.) and fluorescein; slit width = 10 nm.

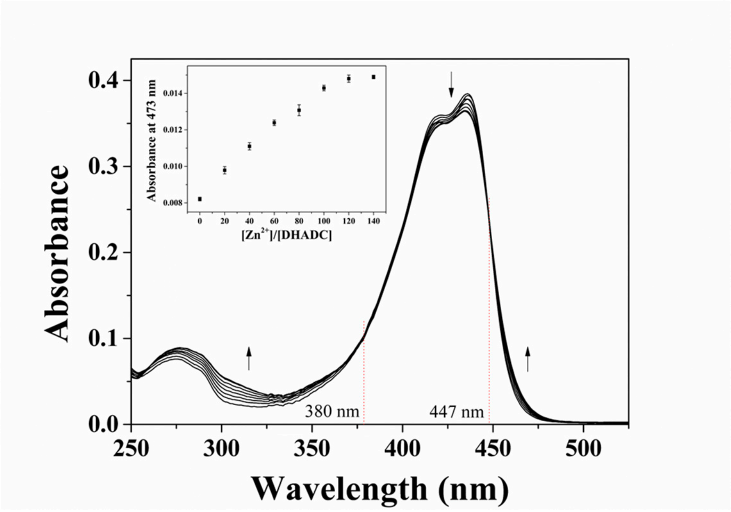

Figure S2. UV-vis absorption spectra of **DHADC** (10 μM) obtained during the titration with $Zn(NO_3)_2$ (0–140 equiv.).

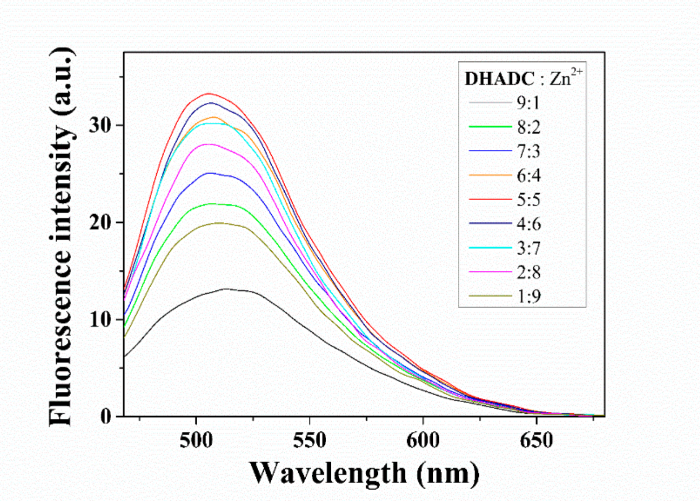

(a)

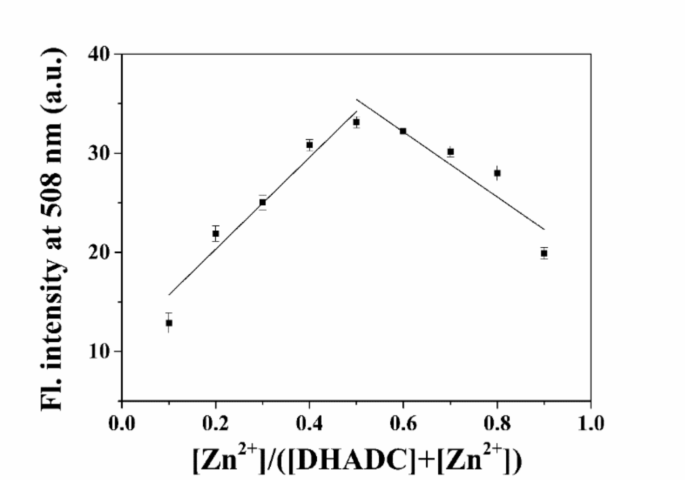

(b)

Figure S3. (**a**) Fluorescence spectra of Job plot for the binding of **DHADC** with Zn^{2+}. (**b**) Intensity at 508 nm was plotted as a function of the molar ratio $[Zn^{2+}]/([$**DHADC**$] + [Zn^{2+}])$. The total concentration of zinc ion with **DHADC** was 7.0×10^{-5} M. λ_{ex} = 446 nm; slit width = 10 nm.

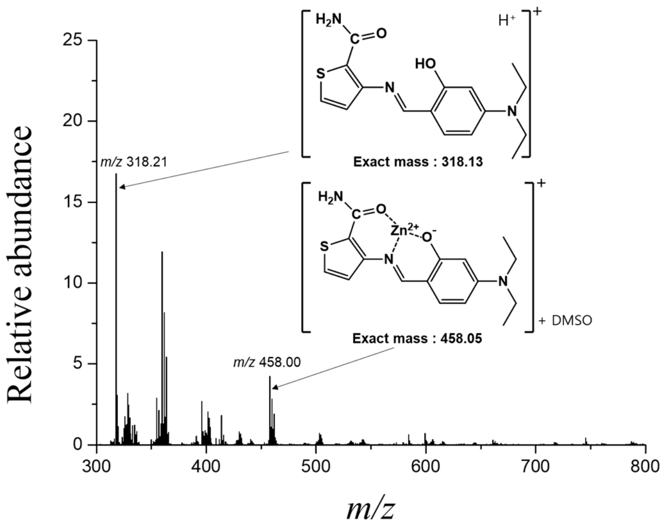

Figure S4. Positive-ion electrospray ionization mass spectrum of **DHADC** (100 μM) upon addition of 1 equiv. of Zn^{2+}.

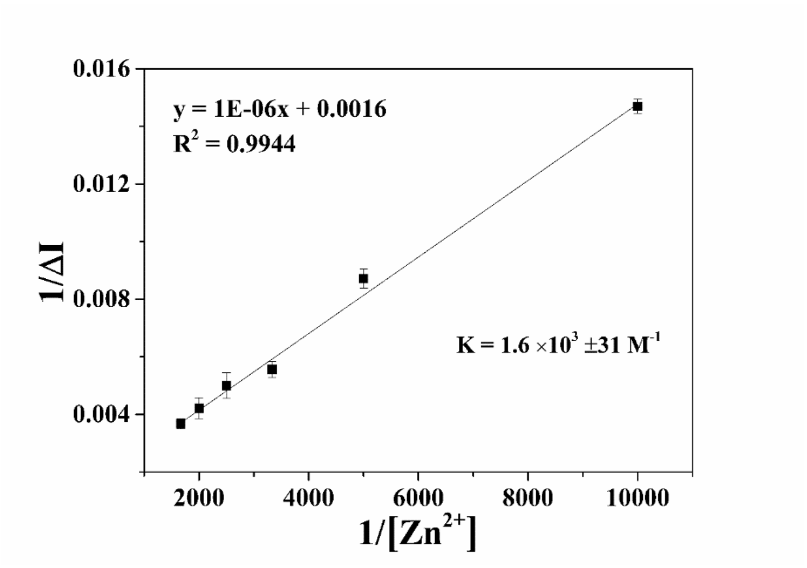

Figure S5. Benesi-Hildebrand equation plot (intensity at 508 nm) of **DHADC**, assuming 1:1 stoichiometry for association between **DHADC** and Zn^{2+}. λ_{ex} = 446 nm; slit width = 10 nm.

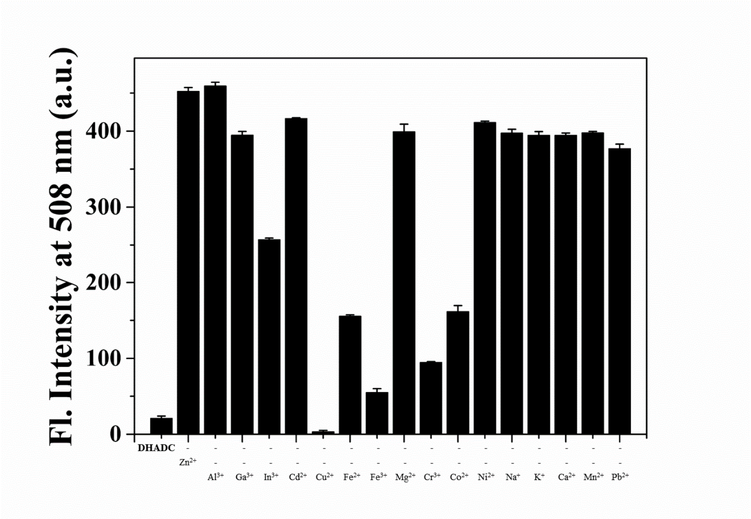

Figure S6. Competitive selectivity of **DHADC** (10 μM) toward Zn^{2+} (140 equiv.) in the presence of other metal ions (140 equiv.). λ_{ex} = 446 nm; slit width = 10 nm.

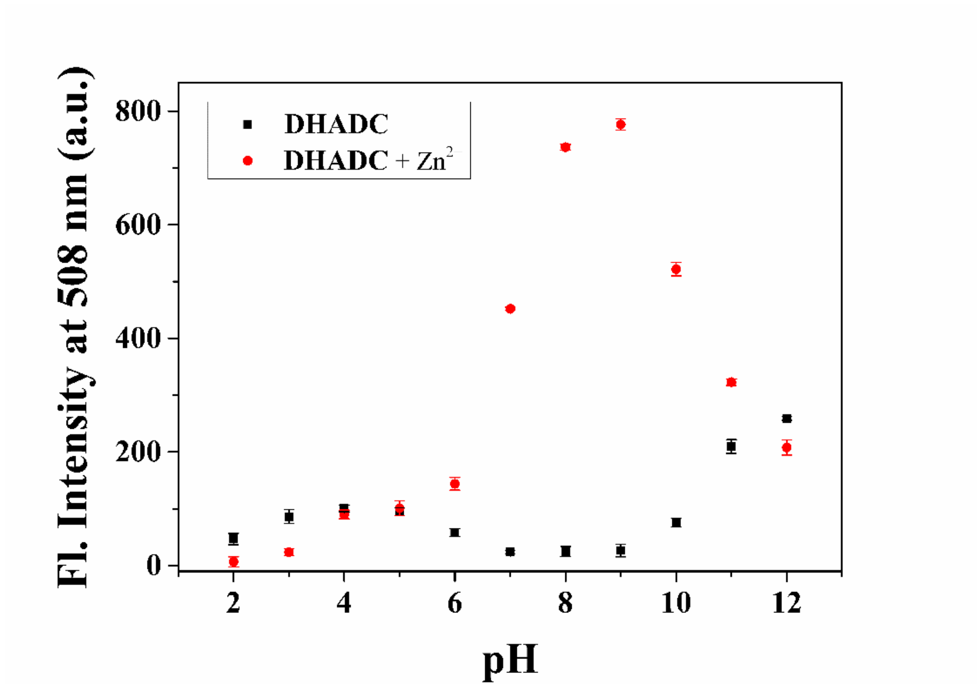

Figure S7. Fluorescence intensities (at 508 nm) of **DHADC** and **DHADC**-Zn^{2+} complex, respectively, at different pH values (2–12). λ_{ex} = 446 nm; slit width = 10 nm.

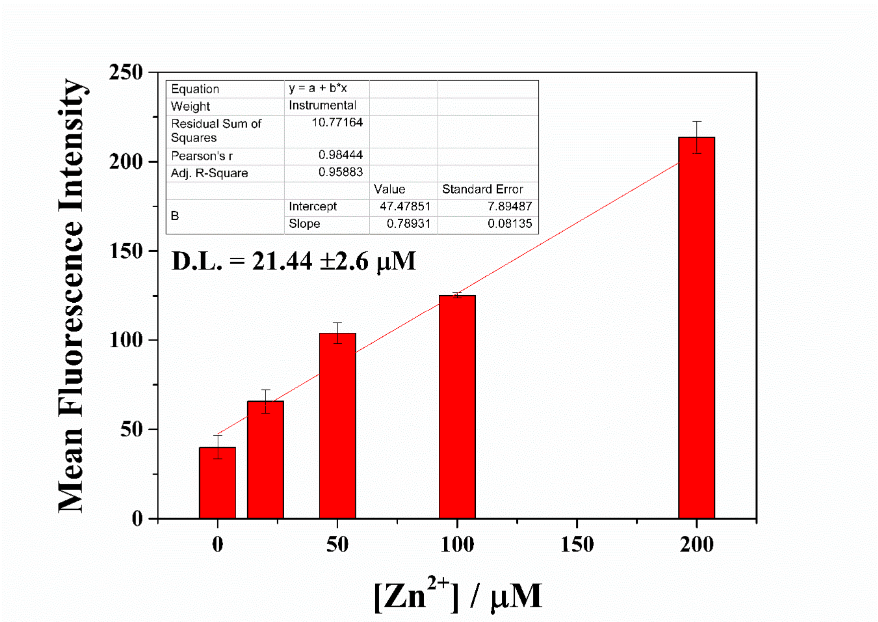

Figure S8. Quantification of mean fluorescence intensity in Figure 6 (**a**$_2$, **b**$_2$, **c**$_2$, **d**$_2$ and **e**$_2$).

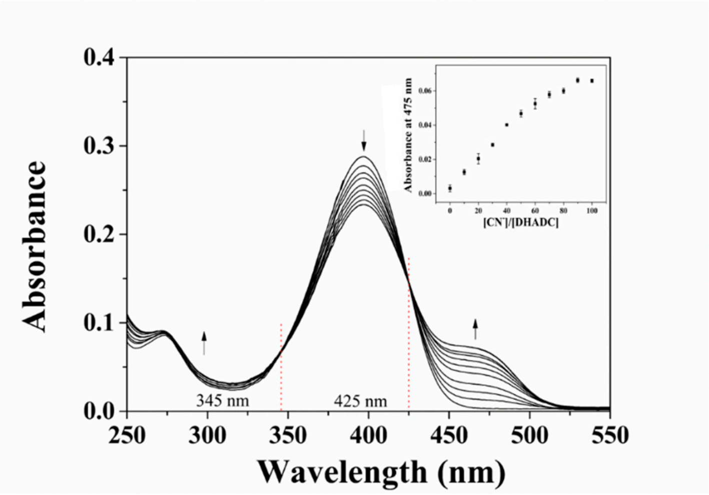

Figure S9. UV-vis absorption spectra of **DHADC** (10 μM) obtained during the titration with CN^- (0–100 equiv.).

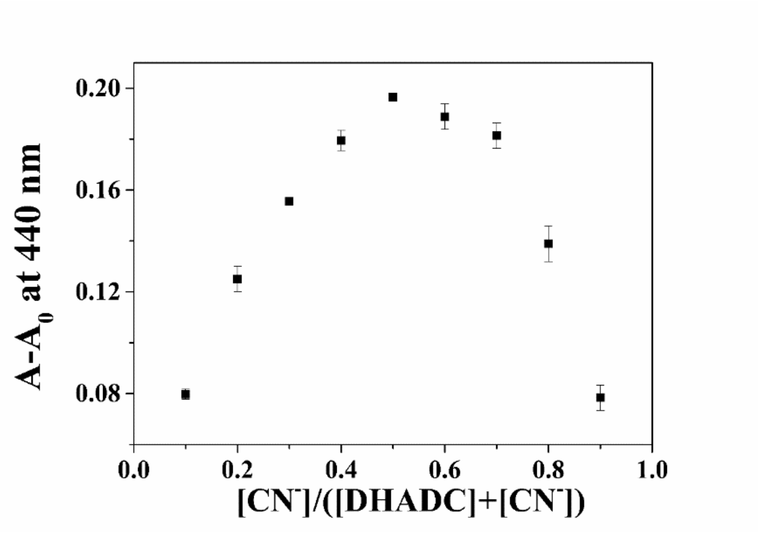

Figure S10. Job plot for the binding of **DHADC** with CN^-. Absorbance at 440 nm was plotted as a function of the molar ratio $[CN^-]/([\mathbf{DHADC}] + [CN^-])$. The total concentration of CN^- with **DHADC** was 2.0×10^{-4} M.

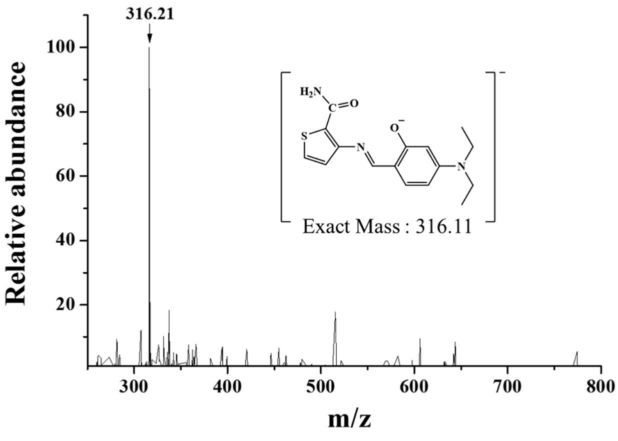

Figure S11. Negative-ion electrospray ionization mass spectrum of **DHADC** (100 μM) upon addition of 1 equiv. of CN^-.

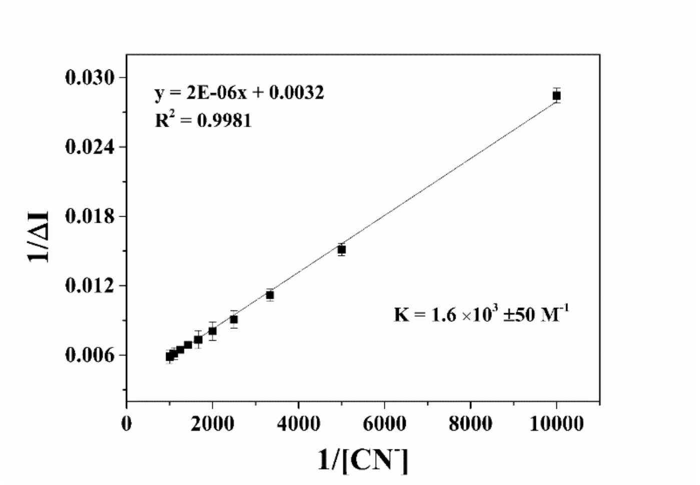

Figure S12. Benesi-Hildebrand equation plot (intensity at 528 nm) of **DHADC**, assuming 1:1 stoichiometry for association between **DHADC** and CN^-. λ_{ex} = 459 nm; slit width = 10 nm.

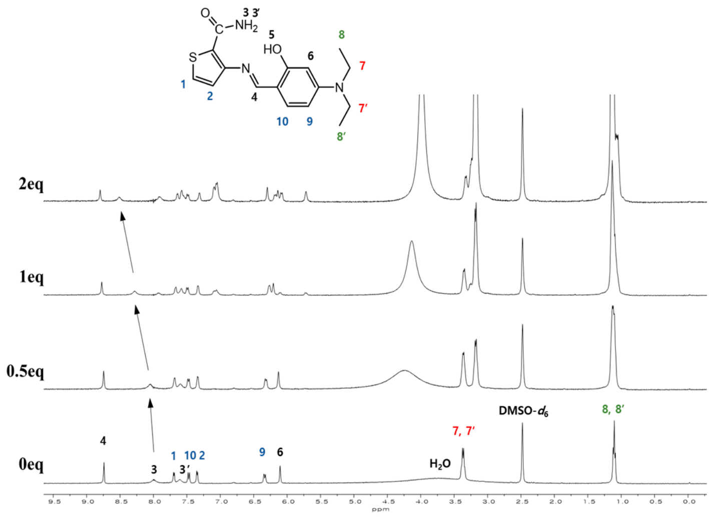

Figure S13. ^{1}H NMR titration of **DHADC** with CN^-.
